# Adherence to antiretroviral therapy among HIV-infected children receiving care at Kilimanjaro Christian Medical Centre (KCMC), Northern Tanzania: A cross- sectional analytical study

**DOI:** 10.11604/pamj.2014.17.238.2280

**Published:** 2014-03-28

**Authors:** Amos Haki Nsheha, Dorothy Elizabeth Dow, Gabriel Erick Kapanda, Bernardus Carolus Hamel, Levina January Msuya

**Affiliations:** 1 Department of Paediatrics and Child Health, Kilimanjaro Christian Medical University College, P. O. Box 2240, Moshi, Tanzania; 2 KCMC- Duke Collaboration, P.O Box 3010, Moshi, Tanzania; 3 Department of Epidemiology & Biostatistics, Kilimanjaro Christian Medical University College, P. O. Box 2240, Moshi, Tanzania; 4 Directorate of Postgraduate Studies, Kilimanjaro Christian Medical University College, P.O Box 2240, Moshi, Tanzania

**Keywords:** HAART, Adherence, Tanzania, HIV, children

## Abstract

**Introduction:**

Paediatric adherence to Highly Active Antiretroviral Therapy (HAART) is a dynamic process involving many factors. Adherence for the majority on therapy matters to prevent failure of 1^st^ and 2^st^ line therapy. The purpose of this study was to determine the rate of adherence to antiretroviral therapy in HIV infected children.

**Methods:**

We conducted a cross-sectional hospital based analytical study, from October 2011 to April 2012. HIV-infected children aged 2 to 17 years who had been on treatment for at least six months were enrolled. Data were collected by a standard questionnaire. Two-day self-report, one month self-recall report, and pill count were used to assess adherence.

**Results:**

One hundred and eighty three respondents participated in this research. There were 92 (51%) males and 91 (49%) females. Only 45 (24.6%) had good adherence to their drug regimen when subjected to all three methods of assessment. Males were more adherent to ART than females (OR= 2.26, CI 1.05-4.87, p = 0.04). Adherence was worse among children who developed ART side effects (OR= 0.19, CI 0.07- 0.56;p = 0.01), could not attend clinic on regular basis (OR= 3.4, CI 1.60- 7.36, p = 0.01) and missed drug doses in the six months period prior to interview (OR= 0.40, CI 0.18-0.82, p= 0.01).

**Conclusion:**

Only 24.6% of paediatric patients had good adherence to ART when subjected to all three measures. Drug side-effects, missing drug doses in the six months period prior to study start, monthly income and affording transportation to the clinicwere strong predictors of adherence.

## Introduction

More than two million children are infected with HIV globally and sub-Saharan Africa bears more than 90% of the global HIV burden [1]. In Tanzania, the HIV epidemic has spread rapidly to all districts and communities and affected all sectors of the society. The estimated HIV prevalence was 5.7% in 2009 equivalent to 1.4 million people; approximately 96,000 died of AIDS in 2008 and only 55.2% of the 425,725 children and adults who qualify forART received treatment [2].

HIV/AIDS requires long-term treatment and adherence to medication is believed to decrease with time. For patients taking daily medication, the exercise is tedious and in six months, compliance to medication was shown to decrease significantly [3].

Adherence requires dedication of both the child and caregivers to consistently follow treatment recommendations [4]. Not only do the child and caregivers need to be committed to treatment, but a variety of other factors may complicate ART adherence in children [5].

With introduction and successful use of Highly Active Antiretroviral Therapy (HAART),HIV-infected children are surviving into adolescence and facing many adherence challenges associated with long-term therapy [5]. Poor adherence increases the risk of virologic failure and viral resistance, therefore, optimal adherence (> 95% of pills taken) is the key to success in HIV infected children who are onlong term treatment [4].

Many developing countries have a high rate of treatment failure among HIV-infected children who are on ART, including those in Tanzania [5]. At KCMC, approximately 20% of 400 children are on second line regimen following failure of the first line regimen.

Children on treatment are surviving longer and reach adolescence, a stage of life which has high risk for engaging in sexual behaviour possibly resulting in transmission of a resistant HIV strain.

The aim of this study was to determine the rate of adherence and factors associated with adherence to antiretroviral therapy among HIV-infected children.

## Methods

This study was conducted at the Child Centred Family Care Clinic (CCFCC) of KCMC, a tertiary hospital, from September 2011 to April 2012. It wasa cross-sectional study thatmeasured adherence levels to ARV drugs and examined demographic and other characteristics of the respondents. The study population included HIV- infected children aged 2 to 17 years on ARVs.

One hundred and eighty three children as well as their caregivers agreed to participate and signed informed consent. Participants were interviewed using a structured questionnaire to obtain demographic characteristics of respondents and subjected to all adherence measurement tools. Three measurements tools were used to assess adherence in children: two-day self-report,one month self-recall report (visual analogue scale) and unannounced pill count.

For the two-day self-report, patients were asked at follow up if they had missed any doses over the last two days. If they reported no missed doses, it was considered good adherence(>95%), while if they missed one dose it was considered poor adherence. For the one- month self-recall (visual analogue scale), the patient was asked to mark in a line to recall the way they took their medicines and thereafter the mark was measured using a 10-centimetre ruler and translated into percentages. If the mark measured below 9.5cm (equivalent to 95%) it was considered good adherence.

By pill count, patients were asked to bring their pill bottles and at random pills were counted. Pill count expectations were derived based on the equation: Number of pills actually taken divided by total number of pills which were supposed to be taken over that period. Anything between 95% and less was considered poor. For fixed-dose combination drugs (Duovir N, Triomune junior -2 pills per day), if more than 2 pills over a one-month period were counted, this was considered poor adherence and if 2 pills or less were counted, it was considered good adherence. For lopinavir based regimen (Didanosine, Abacavir and Alluvia -7 pills per day) which is Tanzanian second line therapy, if more than 8 pills were counted, this was considered poor adherence while if 8 pills or less were counted it was considered good adherence. For Efavirenz based regimen (Combivir and Efavirenz-3 pills per day), if more 4 pills were counted this was considered poor adherence while if ±4 pills were counted it was considered good adherence.

Data generated from the questionnaire was entered in the computer and was analysed using statistical package of social science (SPSS) version 16.0. Descriptive statistics were conducted first to summarize adherence rate measures and socio- demographic characteristics of the respondents. Multivariate analyses were run to find associations between the exposure variables and adherence. All values with a p value of < 0.05 were considered as statistically significant. To control for confounders, all variables with p value < 0.05 were subjected to stepwise logistic regressions.

Ethical clearance was obtained from Kilimanjaro Christian Medical University (KCMU) College Research and Ethics Review Committee and permission to carry out the study was obtained from the Executive Director of the hospital.

## Results

**Demographic characteristics of respondents** (Table 1) Of 183 patients, 92 (51%) were males and 91 (49%) were females. Majority (n = 117; 61.7%) of respondents were adolescents aged 10-17 years and were attending school (n = 160; 87.4%). Most (n = 150; 82.0%) of caregivers were females. Majority (n = 113; 61.7%) of the caregivers were biological parents, had completed primary school (n = 113; 61.7%) and were employed (n = 154; 84.2%).

**Table 1:** Child/caregiver's socio- demographic characteristics (n= 183)

Variable		N (%)
Children		
**Sex**		
	Male	92 (50.3)
	Female	91 (49.7)
**Age group**		
	Pre-adolescent (2-9 years)	66 (36.1)
	Adolescent ( 10-17 years)	117 (63.9)
**Schooling status**		
	Attending school	160 (87.4)
	Not attending school	23 (12.6)
**Caregivers sex**		33 (18.0)
	Male	33(18.0)
	Female	150 (82.0)
**Age group**		
	20 – 29	17 (9.3)
	30 – 39	69 (37.7)
	40 or older	97 (53.0)
**Education level**		
	None	5 (2.8)
	Primary	113 (61.7)
	Post-primary	65 (35.5)
**Marital status**		
	Single	17 (9.3)
	Married	115 (62.8)
	Divorced/separated	15 (8.2)
	Widowed	36 (19.7)
**Relation to the child**		
	Biological parent	113 (61.7)
	Non biological	70 (38.3)
**Occupation**		
	Unemployed	29 (15.8)
	Employed	154 (84.2)
**Average monthly household income (TZS):**		
	Up to 100,000	91 (49.7)
	Above 100,000	92 (50.3)
**Main source of household food:**		
	Purchase	149 (81.4)
	Own source	30 (16.4)
	Support from relatives/friends	4 (2.2)
**HIV status of primary caregiver**		
	Positive	97 (53.0)
	Negative	86 (47.0)

### Antiretroviral drug adherence levels

Adherence level was assessed by three methods. Good adherence was found by two- day self-report in 148 (80.9%), by visual analogue scalein 136 (73.4%), and by pill count in 64 (35%) patients. Only 45 (24.6%) patients had good adherence when subjected to all three measurements.

### Medication and socio-economic factors that influence adherence to ART (Table 2)

Males were more adherent to ART as compared to females (OR = 2.26, CI 1.05-4.87; p = 0.04). Children who were prescribed fixed-dose combination of ARV drugs had better adherence as compared to those prescribed unfixed dose combination(OR = 1.34, CI 0.62-3.08; p= 0.44), though this did not reach statistical significance. Those children who developed drug side-effects were significantly less likely to adhere to medication as compared to those who did not develop drug side effects(OR = 0.19, CI 0.07- 0.56 p = 0.01). Children who missed taking drug doses in six months period prior to interview were also less likely to adhere (OR= 0.40, CI 0.18-0.82; p = 0.01).

**Table 2:** Medication and socio-economic factors that influence adherence to ART

Variable	Total	Adherence status		OR (95% CI)	p-value
		Good adherence	Poor adherence		
		No. (%)	No. (%)		
**Sex**					
Male	92	80 (87.0)	12 (13.0)		
Female	91	68 (74.7)	23 (25.3)	2.26 (1.05-4.87)	0.04
**Age group**					
Pre- adolescents	66	50 (75.8)	16 (24.2)		
Adolescent	117	98 (83.8)	19 (16.2)	0.61 (0.29-1.28)	0.19
**ARV regimen of the child**					
Fixed dose combination	135	111 (82.2)	24 (17.8)		
Unfixed combination	48	37 (77.1)	11(22.9)	1.34(0.62-3.08)	0.438
**Have experienced ARV side-effects**					
Yes	16	8 (50.0)	8 (50.0)	0.19 (0.07-0.56)	0.001
No	167	140 (83.8)	27 (16.2)		
**Missed taking drugs in six months period**					
Yes	61	43 (70.5)	18(29.5)	0.40 (0.18-0.82)	0.012
No	122	105(86.1)	17 (13.9)		
**Average monthly household income (TZS)**					
Up to 100,000	91	68 (74.7)	23 (25.3)		
Above 100,000	92	80 (87.0)	12 (13.0)	0.44 (0.21-0.96)	0.035
**Afford bringing the child to clinic**					
Yes	112	99(84.4)	13 (11.6)	3.4 (1.60-7.36)	0.001
No	71	49 (69.0)	22 (31.0)		
**Providing additional food while the child is taking drugs**					
Yes	152	128(84.2)	24 (15.8)	2.93 (1.23-6.90)	0.011
No	31	20 (64.5)	11 (35.5)		

Children whose caregivers had household monthly income was above 100,000TZS (OR= 0.44, CI 0.21-0.96;p = 0.04) and afforded to transport their children to the clinic (OR= 3.4, CI 1.60-7.36; p = 0.01) were more adherent to ART as compared to those with household monthly income below 100,000TZS and who could not afford to transport their children to clinic on regular basis. Children whose caregivers bought them additional nutritious food were more adherent than those who did not receive additional food (OR = 2.93, CI 1.23-6.90; p = 0.01).

### Predictors of adherence

Multivariate logistic regression was undertaken to determine the predictors of adherence. There were six variables with p values < 0.05: sex, ARV side-effects, missed taking ARV in six months period, monthly household income, affording transportation to the clinic and providing additional food to children taking medication. Of these, only four remained significantly associated with more than 95% adherence to HAART: ARV side effects, missed doses in six months period, household monthly income and affording transportation to the clinic.

## Discussion

This study highlighted an adherence rate of only24.6% in this cohort. Though other studies have shown significantly better results with adherence ratesranging from 69% to 97.5% [6–10], among 159 HIV-positive adolescents of the REACH study, only 28.3% reported complete adherence the previous month by self report [11]. There are several possible explanations for these differences. First, most respondents in this study were adolescents, who had been on ART between 1 year and 5 years as compared to other studies [6–9] in which the studied groups were on treatment for one year. In children on long-term treatment, compliance to medication tends to decrease with time [3]. Second, adolescents in this cohort provided their own account of their adherence as opposed to their caregivers, which was not the case in other studies where the caregivers were providing adherence reports. This only explains the difference when it is assumed that adolescents are less biased and more accurate than caregivers. Third, the adherence rate was obtained using three methods of assessment which was not the case in the other studies: Nabukeera et al used 3 and 7 days self- report and pill count methods [6]; Bhattacharya et al used 4 days recall [7]; Arrive et al used one month self-recall [8];Van Dyke 3 days self-report [9]; Bisimba et.al. 2 day self-report [10]; and Murphy et al. used one month self-recall [11]. To increase sensitivity and reliability of the assessment, more than one measure should be used because they complement each other.

Surprisingly, male children had better adherence than female (OR = 2.26, CI 1.05-4.87; p = 004). This is consistent with the findings reported by Bhattacharyaet al. [7], but inconsistent with that of Nabukeera et al. [6]. The explanation for this is not clear, but it could be explained, partly by cultural norms and taboos. In Tanzania, girls are more engaged in household activities than boys. In the course of accomplishing the given tasks, they might end up tired and hence forget to take their pills, resulting in poor adherence.

Children who were prescribed fixed dose combination ART had better adherence than those prescribed unfixed dose combination, though this did not reach statistical significance. This is in contrast to the findings reported by Nabukeera et al. in Uganda [6] and that of Martin et al. in USA [12] which showed that children who were taking complex regimens had better adherence than those on simple regimens. This was not the case in this study whereby those on unfixed combination were less likely to adhere to medication, possibly because of increased pill burden. Murphy et al. [11] found an inverse relationship between number of drugs and adherences noting more drugs were associated with poorer adherence and simplified regimens may improve adherence.

It was also found that; children who developed ARV drug side effects were less likely to adhere to medication than those who did not develop side effects. This finding is consistent with that reported by Paranthaman et al. in India [13] but inconsistent to the findings reported by Nabukeera et al. in Uganda [6] which showed that drug related factors did not affect adherence. In our study, respondents had been on treatment for more than one year and hence had an increased chance of developing side-effect; however, in the Nabukeera study respondents where only on ART for one year. To minimize the occurrence of drug side effects, more explanations of the drug side effects and how to manage them should be provided to both caregivers and children. Children who missed taking drug doses in the six months period prior to study were less likely to adhere to ART when evaluated by adherence measuring tools in this study, and this six months period history was not noted by other studies in the literature. The study results highlighted those children whose caregivers had good economic status, afforded to bring their children to clinic on regular basis and incurred extra cost for additional food for them; were moreadherent to medication as compared to their counterparts. These findings are similar to those reported by Bhattacharya et al. in New Delhi [7] which showed that children whose caregivers were of low socio-economic status had poor adherence. Empowering family members on income generating activities might raise household income and hence improve adherence.

## Conclusion

In conclusion, only 24.6%of patients had good adherence to ART when subjected to all three adherence measures which is lower than found in many other studies. Statistically significant predictors of poor adherence based on multivariate analysis includedexperiencing ARV drug side effects, having missed drugs doses in six months prior to study period, affording transportation to the clinic and level of household monthly income. To maximize adherence to ARV drugs: it is important to explain drug side-effects and how to manage these side-effects to both caregivers and children. This study also found empowering family members on income generating activities might raise household income and hence improve adherence.

### Limitation

The main limitation of this study is recall bias as well as desirability bias which have been minimized by the-use of objective measures of adherence, pill count method in particular.
